# Strengthening health technology assessment in Greece: industry-identified barriers and recommendations

**DOI:** 10.3389/fpubh.2026.1790128

**Published:** 2026-03-10

**Authors:** Papageorgiou George, Karampli Eleftheria, Athanasakis Kostas

**Affiliations:** 1 School of Public Health, Department of Public Health Policy, University of West Attica, Athens, Greece; 2 Laboratory for Health Technology Assessment, Department of Public Health Policy, School of Public Health, University of West Attica, Athens, Greece

**Keywords:** health policy reforms, health technology assessment, healthcare decision-making, healthcare resources use, stakeholders perspectives, value-based reimbursement

## Abstract

**Objective:**

This qualitative study explores pharmaceutical industry stakeholders’ perspectives on Health Technology Assessment (HTA) in Greece and its perceived implications for public reimbursement decision-making. Since 2018, the Greek Committee for the Evaluation and Reimbursement of Medicines for Human Use has played a central role in recommending medicines for inclusion in the national reimbursement list. Understanding the views of a key HTA stakeholder group provides insight into how the post-2018 HTA and negotiation pathway is experienced in practice and how it is perceived to support timely, evidence-informed decision-making.

**Methods:**

Seventeen senior Market Access executives from pharmaceutical companies were recruited through purposive sampling and interviewed using a semi-structured interview guide. Data were analyzed using thematic analysis, with iterative coding and theme refinement to support analytical rigor.

**Results:**

Nine themes were identified, reflecting participants’ concerns regarding the HTA Committee’s staffing capacity, technical expertise, and transparency. Additional challenges described by participants included legislative misalignment, bureaucratic inefficiencies, limited stakeholder engagement, and delays associated with complex and multi-criteria assessment processes. Additionally, participants proposed fifteen (15) targeted recommendations aimed at strengthening HTA implementation in Greece.

**Conclusion:**

The findings provide stakeholder-informed insights relevant for policymakers seeking to enhance Greece’s HTA framework. As perceived by participants, priorities include strengthening governance and independence, addressing capacity and skills gaps through structured development, clarifying evaluation criteria, increasing transparency, enabling more meaningful stakeholder participation, and advancing digitalization. These improvements have the potential to support more consistent, evidence-informed reimbursement decisions, facilitate timely access to innovative therapies, and contribute to healthcare system sustainability.

## 1 Introduction

The Global Financial Crisis (GFC) of 2007–2008 placed substantial pressure on European Union (EU) economies and public systems, including healthcare, and intensified longstanding challenges in health system financing (1–3). In Greece, fiscal constraints, structural weaknesses, and growing efficiency pressures sharpened the policy need for mechanisms that support evidence-informed prioritization and efficient allocation of healthcare spending (4).

Organization for Economic Co-operation and Development (OECD) data indicate a marked decline in health expenditure per capita during this period, raising concerns about access to services and quality of care in Greece (4). Within this context, Health Technology Assessment (HTA) has been increasingly recognized as a policy tool to support evidence-informed prioritization and to improve efficiency and allocative value in the use of constrained healthcare resources (5, 6). The World Health Organization (WHO) defines HTA as “a systematic approach to evaluate the properties, effects, and impacts of health technologies or interventions” (7). In practical terms, HTA supports decisions on the adoption, reimbursement, and use of health technologies and can be applied across a wide range of interventions, including medicines, medical devices, procedures, health services, and public health interventions (7).

In 2018, Greece established the Committee for the Evaluation and Reimbursement of Medicines for Human Use (hereafter, HTA Committee), alongside the Price Negotiation Committee (Negotiation Committee), to support reimbursement decision-making (8, 9). According to the HTA Committee’s internal regulations, the evaluation considers clinical benefit, comparison with reimbursed alternatives, the robustness of clinical trials, budget impact, and cost-effectiveness (9). In practice, medicines that receive a positive HTA Committee opinion proceed to the Negotiation Committee, where financial terms are negotiated with the Marketing Authorization Holder (MAH) and the expected budget impact is assessed as part of the negotiation process, consistent with the formal “referral to the Negotiation Committee for budget impact assessment” described in the appraisal documentation (8, 9). This sequencing may, however, blur role delineation between technical appraisal and economic negotiation across the reimbursement pathway, given that responsibilities for budget impact considerations are described in both the HTA evaluation criteria and the downstream negotiation process (8, 9).

The Negotiation Committee operates within a wider fiscal environment that includes expenditure caps and mandatory payback arrangements (clawback), under which pharmaceutical companies reimburse the public payer when public pharmaceutical expenditure exceeds the public sector contribution cap (10, 11). Published evidence suggests this cost-containment approach has coincided with price controls and increased patient co-payments, while industry clawback liabilities increased substantially, from approximately €75 million in 2012 to approximately €1.5 billion in 2021 (10, 12). In this context, anticipated clawback exposure can materially shape negotiation dynamics and translate into substantial effective discounts and additional rebates for MAHs beyond list-price reductions (12). Upon completion of negotiations, the Negotiation Committee communicates its recommendation to the HTA Committee, which subsequently issues the final recommendation for reimbursement to the Minister of Health (8, 9). Additional procedural details are provided in Supplementary Material 1.

To date, published analyses of HTA in Greece have primarily focused on legislative design, institutional arrangements, and system-level performance indicators, such as timelines and productivity (13–15). Less attention has been paid to how the post-2018 HTA and negotiation pathway is experienced in practice by stakeholders directly involved in evidence generation, appraisal interactions, and reimbursement negotiations. This study addresses this gap by exploring the pharmaceutical industry’s perspectives on HTA implementation in Greece and identifying actionable opportunities to improve timeliness, transparency, and evidence-based reimbursement decision-making.

## 2 Materials and methods

### 2.1 Purpose of the study

The purpose of this study was to investigate the pharmaceutical industry’s perspective on Health Technology Assessment (ΗΤΑ) in Greece, recognizing that this stakeholder group plays a central role in generating and submitting evidence for appraisal and in engaging with reimbursement-related processes. The study sought to document Market Access executives’ insights, feedback, and lived experiences of the post-2018 HTA and negotiation pathway for medicines, with the aim of characterizing perceived process features and generating stakeholder-informed reflections relevant to reimbursement decision-making, rather than to provide an objective evaluation of system performance.

### 2.2 Study setting

The website of the Hellenic Association of Pharmaceutical Companies (SfEE) was used to identify pharmaceutical companies operating in Greece through its publicly available membership list.^1^ SfEE is an industry body representing the majority of pharmaceutical companies active in the Greek market, and its publicly available membership list provided a centralized and up-to-date source for mapping potential participants. No empirical data were collected from SfEE; the organization served solely as a means of identifying member companies for recruitment purposes. SfEE encompasses 59 member companies, including both Greek-owned pharmaceutical firms and multinational companies.^2^

### 2.3 Description of the participants

Eligible participants were selected using purposive sampling, a technique in which individuals are intentionally chosen based on specific characteristics relevant to the research objectives (16, 17). Inclusion criteria were: (i) Senior Market Access role (Department Heads or Directors), (ii) active involvement in pricing, HTA submission/appraisal interactions, reimbursement, and negotiation processes in Greece, and (iii) affiliation with a member company of SfEE. Executives without direct responsibility for market access activities in Greece or without recent experience with HTA/negotiation processes were excluded. This approach ensures that participants with the most pertinent expertise and insights are included, thereby enhancing the depth and relevance of the study (16, 17).

An anonymized summary of participant characteristics Table 1.

**Table 1:** Profile of participating market access executives.

Anonymized participant characteristics	
Total participants	17
Seniority level	Heads/Directors of Market Access
Years of professional experience	<5 (*n* = 1) 5–10 (*n* = 2) 10–15 (*n* = 6) > 15 (*n* = 8)
Involvement in HTA and negotiation processes	All participants
Type of organization	Pharmaceutical companies operating in Greece

Participants were approached through professional networks and direct outreach to Market Access leads in SfEE member companies. Snowball sampling was used subsequently, whereby interviewed participants suggested additional eligible executives, who were then contacted by the research team (17). This sampling strategy aimed to capture a range of perspectives across different company types, including Greek-owned and multinational pharmaceutical firms.

### 2.4 Data collection

Qualitative techniques, specifically semi-structured interviews, were employed to gather in-depth insights into the perspectives of the participants. Semi-structured interviews are advantageous for their flexibility and depth, allowing for the exploration of complex issues while providing a structured framework to ensure consistency across interviews (16, 18). These interviews were guided by a structured interview guide, which consisted of eight main questions designed to cover the key areas of interest while allowing for in-depth exploration of each topic (19). The interview guide ensured that all interviews addressed the same core issues, facilitating comparative analysis and ensuring comprehensiveness (19).

The interview guide can be found in Supplementary material.

To refine the interview questions and ensure clarity, *pilot* interviews were conducted. Two executives, who met the inclusion criteria, were approached to participate in these pilot interviews (19). During the first pilot, issues were identified in two of the eight questions, which were subsequently rephrased for clarity. No further issues arose during the second pilot interview, indicating the refinement process was successful. All interviews were conducted following informed consent procedures, as detailed below.

### 2.5 Data analysis

Thematic analysis was utilized to analyze the interview transcripts, a method known for identifying and analyzing recurring patterns and themes within qualitative data (20, 21). Thematic analysis is a flexible method that allows for both a rich description of the data and a deep understanding of the patterns that emerge (20, 21). This approach involves systematically coding the data, identifying themes, and then interpreting these themes to gain insights into the participants (20, 21).

The analysis incorporated both inductive and deductive approaches to thoroughly examine and understand the participants’ viewpoints (20, 22). Inductive analysis involves deriving themes from the data itself, while deductive analysis applies existing theories or concepts to the data (20, 22). The first five interview transcripts were independently coded by two researchers to support interpretive rigor and to develop an initial coding framework. Codes were compared and discussed, and areas of divergence were resolved through consensus. The remaining transcripts were subsequently analyzed by the main researcher using this framework, with iterative refinement as analysis progressed. Codes were then grouped into higher-order themes and subthemes based on conceptual similarity and content, with themes reviewed collaboratively for internal coherence and conceptual distinction.

Given the researcher’s professional familiarity with health policy, medicines reimbursement, and market access processes in Greece, reflexive notes were maintained throughout data collection and analysis to document emerging assumptions and support critical reflection during interpretation.

### 2.6 Data retention

Audio files and transcripts were stored on encrypted, password-protected devices/servers with access restricted to the research team. Raw study data (audio recordings and transcripts) will be retained for 5 years after completion of data analysis and then securely deleted.

### 2.7 Ethics and consent

Written informed consent was obtained from all participants via electronic signature prior to participation, including consent for audio-recording of the interviews (23). As pseudonymity helps to protect participant identities and mitigate the risk of personal data exposure, confidentiality was protected using pseudonyms (e.g., Participant 1, Participant 2) (24). In addition, all data were encrypted and stored to ensure that sensitive information was safeguarded (24). Participants were fully informed of these measures to support transparency and trust (25). No sensitive personal data were collected beyond participants’ professional role and whether their company was Greek-owned or multinational. In qualitative research, ensuring confidentiality and securing data are essential for maintaining participant trust and the integrity of the research process (18, 21).

Ethical approval was obtained from the Research Ethics Committee of the Greek National School of Public Health following submission and approval of the study protocol (Protocol No: 10802/11-02-2022).

### 2.8 Conducting the interviews

The interviews were conducted between mid-2022 and mid-2023. They were performed online using electronic platforms such as Teams and Zoom. The interviews were recorded following the participants’ informed consent and were subsequently transcribed verbatim by the same researcher, who conducted all interviews. Each interview lasted approximately 1 h. Data saturation was determined as the point at which no new themes or insights emerged from additional interviews (26, 27).

Data saturation was reached by the end of the 17th interview, after which it was concluded that all relevant data had been collected and further interviews would not yield additional information (28). This conclusion was reached collaboratively by all researchers involved in the study, who agreed that the data collected adequately represented the range of perspectives needed for comprehensive analysis.

*The study was reported in accordance with the Standards for Reporting Qualitative Research (SRQR), with additional methodological detail aligned with the Consolidated Criteria for Reporting Qualitative Research (COREQ) for interview-based qualitative studies (**25*, *29**).*

A completed COREQ checklist is provided in the Supplementary material.

## 3 Results

Seventeen executives meeting the inclusion criteria participated in the study. No further interviews were conducted as data saturation was reached. Thematic analysis identified nine overarching themes.

An overview of themes and sub-themes is presented in Table 2.

**Table 2:** Conceptual framework of study themes and sub-themes.

Conceptual framework of study themes and sub-themes				
Health Technology Assessment (HTA) organizational structure				
Sub-themes	Staffing	Knowledge, Expertise and Conflict-of-interests	Delays	Prospect of establishing a Health Technology Assessment Organization
HTA methodology and criteria				
Sub-themes	Criteria completeness		External criteria	
HTA committee’s work in Greece				
Sub-themes	Design, preparation, and deliberation before Law 4512/2018	Executives’ expectations prior to implementation	Satisfaction with evaluation procedures	Need for digitization
Publicity rules and existing legal framework				
Sub-themes	Transparency	Appraisal documentation	Legal framework	Interaction with stakeholders
Role of patients in HTA				
Sub-themes	Patient involvement and the role of patient associations			
Interactions between the HTA committee and the negotiation committee				
Sub-themes	Cooperation framework		Rewarding pharmaceutical innovation	
The executives’ recommendations				
Sub-themes	Fifteen (15) recommendations			
Supplementary material themes				
The role of associations of pharmaceutical companies in health technology assessment				
European Union (EU) joint clinical assessments				
Sub-themes	The adoption of a common European framework for clinical evaluations		Necessity of a national assessment framework	

HTA, Health technology assessment; HTA committee, Committee for the evaluation and reimbursement of medicines for human use (in Greece); Negotiation committee, Price negotiation committee (in Greece); EU, European Union.

Themes 1–7, which capture the main lessons learned regarding the implementation and functioning of the Committee for the Evaluation and Reimbursement of Medicines for Human Use (HTA Committee) and its interface with the Pricing Negotiation Committee (Negotiation Committee), are presented in this paper. Themes 8 and 9, which address (i) the role of associations of pharmaceutical companies in HTA and (ii) European Union (EU) Joint Clinical Assessments, are presented in Supplementary material.

Indicative participant quotes are provided throughout the text.

### 3.1 Theme 1: HTA committee organizational structure

Participants described organizational structure as a central determinant of how the HTA pathway functions in practice. The most frequently discussed issues related to staffing adequacy, expertise and training, conflict-of-interest constraints, and perceived delays.

#### 3.1.1 Staffing

Many respondents perceived that the HTA Committee began its operation under-staffed, including both Committee members and administrative support. Although several acknowledged improvements since its establishment in 2018, the prevailing view was that resources remain insufficient relative to workload.

As Participant 15 stated: “The staff is not sufficient, 11 members with some secretaries who are not really who they should be.”

#### 3.1.2 Knowledge, expertise, and conflict of interest

Many participants expressed concerns about limited training and a perceived lack of prior experience among members, given the complexity of HTA work. Several emphasized the need for stronger health economics expertise, describing assessments as overly clinical in orientation.

Participant 13 stated: “It is a committee with expertise in the pharmacology and with no technical capacity to evaluate whether a drug is cost-effective.”

Participants also consistently argued that the current conflict-of-interest framework creates substantial barriers to recruiting experienced professionals.

Participant 2 stated: “There are too many conflicts of interest, so it’s very difficult to find people not related to industry.”

#### 3.1.3 Delays

Most executives reported delays in the evaluation process and linked these to workload and administrative bottlenecks. A minority noted faster timelines, attributing improvements to leadership and internal coordination.

Participant 4 stated: “I think it has improved over the years. The current President is moving quickly on schedule.”

#### 3.1.4 Prospect of establishing an independent HTA organization

Many respondents considered the transition from a committee-based model to a more independent HTA organization important, emphasizing the need for a clear framework, resources, and planning capacity. However, several expressed skepticism about the feasibility or political commitment to such a transition.

Participant 5 stated: “I never expect it to become an organization. Because Greece operates reactively. If the system crashes because of this process, they can review it. As long as it works as lamely as it does, they won’t mind.”

*Participant quotations are presented verbatim to ensure an accurate representation of participants’ language and perspectives. Such expressions illustrate the strength of participants’ perceptions and should be interpreted as reflecting stakeholder frustration rather than an objective assessment of policy intent or institutional performance.*

A small number expressed reservations about transition, citing potential risks of additional costs and delays.

### 3.2 Theme 2: HTA methodology and criteria

Participants focused on how HTA criteria are applied in practice and whether evaluation requirements are consistently operationalized.

#### 3.2.1 Criteria completeness

Most participants expressed the view that evaluation criteria are not consistently met in practice. Some described limited feedback or documentation demonstrating how submitted evidence is reviewed and incorporated into decisions.

Participant 9 stated: “No one examines what we submit. And if I’m wrong and somebody looks at it, I’ve never seen it, I’ve never got any response, or any documentation.”

One participant suggested that some required documents may not be actively evaluated, reporting that they stopped submitting certain elements without receiving a notification that the dossier was incomplete.

#### 3.2.2 External criteria

In the Greek HTA pathway, “external criteria” refer to an eligibility prerequisite linked to prior reimbursement status in a predefined set of reference countries, commonly described as a “5/11” requirement. In practice, to submit a dossier for evaluation, the MAH must demonstrate that the medicine has been reimbursed in at least five out of the predefined 11 countries, as described in the legal framework governing evaluation criteria and methodology (Hellenic (30)).

Additional procedural details are provided in Supplementary material.

Participants were unanimous that external criteria should be removed, describing them as a source of delay and as misaligned with a structured, evidence-based assessment process. Concerns were also raised about the implicit weight of external criteria in decision-making.

Participant 3 stated: “The decisive factor for the positive evaluation is the external criteria. The system relies on an external reporting system rather than an internal assessment.”

### 3.3 Theme 3: HTA Committee functioning in Greece

This theme captured perceptions of the introduction of the HTA Committee, expectations prior to implementation, satisfaction with procedures, and the perceived need for system digitalization.

#### 3.3.1 Design, preparation, and deliberation before law 4512/2018

Several executives described the implementation as rapid and insufficiently deliberative, with limited engagement of experts and stakeholders during design.

Participant 4 stated: “It was not very consensual. There was no open dialogue, to get the opinion of experts.”

#### 3.3.2 Executives’ expectations prior to implementation

Many respondents reported low expectations prior to implementation, drawing on prior experiences of health system reforms in Greece.

Participant 5 stated: “I expected exactly what happened. And I was even saying it to some in favor of it, that guys, can you imagine a Greek HTA? Few people, some with HTA-related expertise, others without, with a small capacity and a large workload.”

A minority indicated that other foundational elements, such as protocols and registries, should have been addressed before establishing the HTA Committee.

#### 3.3.3 Satisfaction with evaluation procedures

The majority described dissatisfaction with evaluation procedures, citing limited transparency, delays, lack of guidelines, absence of tracking systems, minimal interaction with stakeholders, and approaches perceived as overly cost-oriented without adequately capturing therapeutic value.

Participant 11 stated: “The HTA in Greece is just a tick in the box.”

*Participant quotations are presented verbatim to ensure an accurate representation of participants’ language and perspectives. This expression was used by participant 11 to convey the perception that HTA functions as a formal procedural requirement rather than a substantive evaluative process influencing reimbursement decisions.*

A few respondents reported greater satisfaction, indicating that the HTA Committee represented a positive step compared with the pre-2018 environment.

#### 3.3.4 Need for digitization

Participants consistently emphasized the absence of digitization and the lack of an online platform enabling tracking of submissions and process stages.

Participant 13 stated: “There is no tracking system that says: this is the number of files submitted and completed. The only update we have from the HTA Committee is when the HTA Committee’s President gives an update at the conferences.”

### 3.4 Theme 4: publicity rules and existing legal framework

Participants described transparency and the legal framework as interlinked determinants of how the HTA Committee’s work is perceived and understood.

#### 3.4.1 Transparency

Participants consistently described limited transparency, citing the absence of interaction with companies during evaluation, the lack of a formal objection process, and insufficient procedural guidance (e.g., protocols, guidelines).

Participant 12 stated: “I think that the HTA Committee’s work as it is today, cannot be evaluated because it is a black box.”

#### 3.4.2 Appraisal documentation

Participants consistently perceived appraisals as too brief to support understanding of the rationale and methodological basis of decisions, including limited visibility on analytical approaches.

Participant 12 stated: “It does not make sense to give explanations the way they give it, with the words ‘referred to’ the Negotiation Committee. This is not an appraisal.”

Many respondents also indicated that appraisals communicated to the Negotiation Committee may differ from what companies receive, leaving them insufficiently prepared for subsequent negotiations. Some further noted that appraisal content may largely reproduce submitted information, with limited added interpretation.

#### 3.4.3 Legal framework

Many executives attributed transparency limitations to the legislative framework, emphasizing that it does not provide sufficiently detailed procedural rules for HTA Committee operations.

Participant 3 stated: “The committee operates under the existing legal framework, which is responsible for not ensuring transparency.”

#### 3.4.4 Interaction with stakeholders

Many participants highlighted limited dialogue and cooperation with the HTA Committee.

Participant 3 stated: “The big problem is that the HTA Committee works without cooperating with the companies. So, we don’t have a dialogue, we have the HTA Committee’s monologue.”

### 3.5 Theme 5: role of patients in HTA

This theme reflects perceptions of patient involvement and the role of patient associations in the evaluation process.

#### 3.5.1 Patient involvement and the role of patient associations

Participants consistently noted that patient participation is described vaguely in legislation and that meaningful involvement is important because patients can contribute experiential knowledge and insights on unmet need and real-world impact.

Participant 3 stated: “We need to know if the existing treatments have an effect or not, what is the unmet need.”

While most interviewees supported patient involvement, several suggested that patients should contribute to a consultative role rather than hold a decisive role in appraisal, citing the technical complexity of HTA decisions.

### 3.6 Theme 6: interactions between the HTA committee and the negotiation committee

This theme captured perceptions of coordination, the transfer of information between committees, and the perceived implications for innovation and value recognition.

#### 3.6.1 Cooperation framework

Participants unanimously reported a lack of a structured cooperation framework between the HTA Committee and the Negotiation Committee. Many perceived that HTA appraisals have limited influence on negotiation outcomes, and that the committees’ roles are not complementary in practice.

Participant 4 stated: “The problem starts with the fact that the committees are not supplementary.”

#### 3.6.2 Rewarding pharmaceutical innovation

Participants consistently argued that the current system does not adequately reward innovation. Reported reasons included the absence of a clear operational definition of innovation and negotiation practices perceived as primarily cost-containment focused, with limited differentiation across medicines.

Participant 13 stated: “You are all the same,” says the Negotiation Committee, which then proceeds to the discounting process.

### 3.7 Theme 7: executives’ recommendations

Based on the needs identified across themes, participants proposed fifteen (15) recommendations intended to support a more effective and sustainable HTA system in Greece.

Table 3: *Bridging Gaps: From Identified Needs to Actionable Solutions* summarizes the linkage between perceived needs and proposed recommendatio*ns.*

**Table 3:** Bridging gaps: from identified needs to actionable.

Number of recommendations	Participant-identified needs	Recommendations
1	No participation of stakeholders (Pharmaceutical industry, pharmaceutical associations, academic institutions, patient organizations, medical societies etc.) in the Health Technology Assessment (HTA) decisions.	Active participation of all stakeholders in HTA processes
	Lack of dialogue, cooperation, and interaction with the HTA Committee.	
2	Digitation is the cornerstone of the HTA processes.	Creation of a digital platform for monitoring the progress of an application.
3	Patients cannot participate in the committee with a decisive role because they lack knowledge and experience.	Training of all involved stakeholders
	Absence of continuous trainings of the committee on Health Technology Assessment.	
4	External criteria cause delays and cannot co-exist with a structured evaluation system.	Removal of external criteria
5	Need for better planning and foresight in HTA processes.	Horizon scanning utilization
6	Current conflict-of-interest rules create barriers to attracting experienced executives.	Flexibility in engaging external experts
7	Absence of expertise in health economics among the HTA Committee’s members.	Attracting experts with diverse backgrounds such as health economics
8	Rapid implementation of the HTA committee in Greece with underqualified personnel and rushed decisions.	Establishment of an independent HTA Organization
	No connection between the clinical value of products and their reimbursed price.	
	Delays in the evaluation process due to heavy workload and bureaucracy.	
	HTA Committee is understaffed.	
9	Lack of clear rules in the existing legislative framework.	Guidelines adoption for the whole process
10	Lack of dialogue, cooperation, and interaction with the HTA Committee.	Consultation and interaction with the ΗΤΑ Committee
11	Evaluation criteria are not consistently met or well-described.	Review of the HTA’s requiring supporting documents (criteria)
12	Insufficient number of staff (administrative and evaluators etc.) in HTA Committee and absence of periodic and ongoing trainings.	Strengthening Human Resources
13	Lack of structured cooperation between the HTA Committee and Negotiation Committee.	Participation of an HTA Committee representative in the Negotiation Committee process
14	Greece does not reward pharmaceutical innovation adequately.	Pricing and discounts based on value and novelty
	Ineffective cost-oriented strategies undermine treatment value.	
15	Poor transparency in the procedures and appraisals.	Establishment of a comprehensive publicity process

HTA, Health technology assessment; HTA committee, Committee for the evaluation and reimbursement of medicines for human use (in Greece); Negotiation committee, Price negotiation committee (in Greece).

#### 3.7.1 Fifteen recommendations

1. Active participation of all stakeholders in HTA processes: Establish structured consultation points involving patients, clinicians, academia, HTA experts, and MAHs.
2. Creation of a digital platform for monitoring the progress of an application: Enable real-time tracking of milestones, timelines, and status updates throughout the appraisal pathway.
3. Training of all involved stakeholders: Introduce periodic training on HTA methods, economic evaluation, and appraisal standards for committee members and contributors (HTA personnel, industry representatives, patient associations, Healthcare Professionals, Academics, External experts etc.).
4. Removal of external criteria: Eliminate the ‘external criteria 5/11’ prerequisite, which participants perceived as a major driver of delays.
5. Utilization of horizon scanning techniques: Develop horizon scanning to anticipate upcoming innovations and support prioritization and planning of assessments.
6. Flexibility in engaging external experts: Allow ad hoc engagement of independent specialists to support complex assessments.
7. Attracting experts with diverse backgrounds, including health economics: Strengthen multidisciplinary capacity by recruiting expertise beyond clinical disciplines, particularly in health economics.
8. Establishment of an independent HTA organization: Transition from a committee model toward a more independent structure with defined responsibilities, resources, and planning capacity.
9. Adoption of guidelines for the whole process: Adopt and operationalize clear procedural guidance for submissions, appraisal methods, deliberation, and decision documentation.
10. Consultation and interaction with the HTA Committee: Introduce structured dialogue with MAHs during assessment to address clarification needs and improve appraisal quality.
11. Review of supporting documents and submission requirements: Streamline dossier requirements and ensure each requested document is meaningfully assessed and referenced in the appraisal.
12. Strengthening human resources: Expand administrative and scientific staffing to match workload and reduce bottlenecks and procedural delays.
13. Participation of an HTA Committee representative in the negotiation committee process: Improve continuity by ensuring HTA appraisal outputs and clinical value considerations inform downstream negotiation.
14. Discounts and prices based on value and novelty: Align negotiated financial arrangements with demonstrated clinical value and novelty, rather than uniform discounting practices.
15. Establishment of a comprehensive publicity process: Increase transparency through documentation of stages, timelines, deliberations, and decision rationales across the pathway.

## 4 Discussion

The study highlights several aspects of the evaluation process within the Greek Health Technology Assessment (HTA) framework, as perceived and reported by pharmaceutical industry participants, and identifies challenges and opportunities for improvement. The findings indicate that participants perceived the Committee for the Evaluation and Reimbursement of Medicines for Human Use (HTA Committee) in Greece as facing major challenges related to organizational structure and staffing. Despite reported improvements, participants described a need for a more robust staffing model and sustained capacity-building to strengthen the quality and consistency of HTA evaluations. Similar capacity constraints, particularly around human resources, have been identified as common barriers to effective HTA functioning in other settings (31).

Interviewees emphasized the importance of periodic and ongoing training for the HTA Committee and the need to attract experts in health economics. The importance of rigorous economic evaluation as a core component of HTA is widely recognized, including the value of specialized expertise (41). Consistent with this, World Health Organization (WHO) operational guidance on institutionalizing HTA highlights the contribution of a multidisciplinary composition (e.g., health economists, biostatisticians, clinicians, pharmacists) to strengthening committee-based assessment processes (41).

The transition from a committee-based model to a more independent organizational structure was considered by most participants to be an important prospective reform. In the Greek context, proposals have been put forward that describe prerequisites for a sustainable HTA function, such as planning capacity, staffing, clearer responsibilities, and dedicated resources; yet these proposals have not been operationalized into an independent agency model to date (13).

Securing and safeguarding resources can materially influence the establishment and functioning of HTA arrangements. Evidence from Kenya, for example, underscores how the availability of experienced personnel, financial resources, and organizational structures can facilitate the institutionalization of HTA, while limited resources can undermine it (32).

Regarding process rules and communication, many executives perceived limited transparency, while others attributed this to the legislative framework. Uncertainty about process stages and decision rationales aligns with broader evidence showing that governance arrangements, clarity of process, and institutional frameworks can act as both facilitators and barriers to introducing and sustaining HTA (33). Concerning the completeness and application of HTA criteria, participants reported inconsistencies that may obstruct assessment effectiveness. This resonates with international evidence indicating strong cross-agency convergence on core criteria (e.g., clinical effectiveness, safety, cost-effectiveness, budget impact), while also emphasizing the importance of consistent operationalization of these criteria in real-world decision-making (34).

Participants also identified the use of additional “external” criteria as a source of delay and perceived misalignment with a structured evaluation process. Cross-country evidence indicates that European reimbursement systems vary considerably in how they combine HTA evidence with additional policy criteria and procedural requirements, and that such heterogeneity can introduce complexity into appraisal pathways (35).

Concerns were also raised regarding planning and prioritization, including perceived limited trust and involvement. Participants suggested implementing a digital platform enabling Marketing Authorization Holders (MAHs) to track their application progress and understand prioritization decisions. Good-practice guidance emphasizes that consultation, deliberation, and structured dialogue across stakeholders can support legitimacy, transparency, and trust in HTA processes (36). In addition, transparency and completeness of assessment documentation are frequently highlighted as elements that can strengthen the credibility of HTA outputs; published checklists and guidance for HTA reporting provide structured expectations for what a transparent HTA report should contain (37).

Conflict-of-interest rules were described as restricting the pool of potential HTA Committee members and external evaluators. Participants suggested that revisiting these rules, potentially drawing on established approaches used in mature HTA settings, can facilitate recruitment while maintaining appropriate safeguards (38).

Limited stakeholder participation was also emphasized. Participants acknowledged the value of incorporating patient perspectives, while noting that the current framework does not fully facilitate such involvement. The literature supports the view that patient engagement can enhance relevance, legitimacy, and the alignment of HTA with patient priorities (39). Participants consistently emphasized that, despite formal references in legislation, patient involvement in the Greek HTA process remains largely absent in practice. They highlighted the value of patient input in capturing experiential evidence, unmet need, and quality-of-life considerations, while generally supporting a consultative rather than a decision-making role, particularly at the current stage of system maturity. Concerns were also raised regarding preparedness, training, and potential conflicts of interest among patient representatives. Comparative European experience indicates that patient participation in HTA is commonly structured through consultative mechanisms, written submissions, or participation without voting rights, supported by clear role definitions and targeted capacity-building. These models illustrate potential pathways for strengthening patient involvement in Greece, while underscoring the importance of governance safeguards and training to ensure meaningful and credible contribution (40).

Finally, the study underscores the need for better coordination between the HTA Committee and the Negotiation Committee. Participants perceived that the absence of a clearly structured cooperation framework can limit the influence of HTA outputs on downstream reimbursement arrangements. Cross-country mapping suggests that while HTA commonly informs reimbursement decisions, the weight of HTA evidence in final decisions varies across systems, reflecting differences in institutional design and policy integration (40). Overall, these findings reflect participants’ concerns regarding the strengthening of links between HTA, negotiation, and policy frameworks, highlighting perceived implementation challenges related to timely access and consistent, evidence-based decision-making.

### 4.1 Strengths and limitations

This study has limitations that should be considered when interpreting its findings. First, the analysis is based exclusively on the perspectives of pharmaceutical industry stakeholders. While these participants play a central role in the generation, submission, and negotiation of evidence within the Health Technology Assessment (HTA) process, their views may be influenced by organizational priorities, professional roles, regulatory experiences, and strategic considerations related to market access and reimbursement. As such, the findings reflect stakeholder-specific perceptions of how the HTA and negotiation pathway operates in practice, rather than an objective or comprehensive evaluation of the performance or system-wide effectiveness of the Committee for the Evaluation and Reimbursement of Medicines for Human Use (HTA Committee).

In addition, although the sample size was sufficient to achieve thematic saturation within this stakeholder group, the transferability of the findings to other actors involved in the HTA process—such as policymakers, evaluators, clinicians, or patient representatives—is inherently limited. Future research incorporating multiple stakeholder perspectives could provide a more holistic understanding of HTA implementation dynamics in Greece.

Despite these limitations, the study offers policy-relevant insights into how the post-2018 HTA and negotiation framework is operationalized in practice, particularly during the early phase following the introduction of a formal HTA process. By documenting implementation challenges and system frictions as perceived by stakeholders directly involved in evidence generation, appraisal interactions, and reimbursement negotiations, the findings may also inform other health systems undergoing similar transitions toward institutionalized HTA frameworks.

## 5 Conclusion

This study provides an in-depth qualitative account of challenges currently confronting Greece’s post-2018 Health Technology Assessment (HTA) pathway for medicines, as perceived and experienced by pharmaceutical industry stakeholders. The findings suggest that, from the perspective of participants, strengthening the system would require reforms across governance, capacity, transparency, and stakeholder engagement. Participants indicated that establishing a more independent HTA organizational model could enhance the perceived robustness, consistency, and credibility of evaluations and support clearer delineation between technical appraisal and downstream price negotiation. Addressing staffing constraints and investing in targeted capacity-building, particularly in health economics and appraisal expertise, were described as essential for improving the perceived quality and timeliness of assessments.

In parallel, participants emphasized that more structured and meaningful engagement with stakeholders, including patient associations, clinical societies, academia, HTA experts, and the pharmaceutical industry, could improve inclusiveness and legitimacy. Clearer procedural guidance, greater transparency around evaluation stages and decision rationales, and digital tools enabling stakeholders to track application progress may further strengthen efficiency and trust. Overall, these improvements were perceived by participants as having the potential to support reimbursement decisions informed by more consistent evidence appraisal and aligned with value, thereby facilitating timely access to effective innovations while safeguarding healthcare system sustainability.

Future research integrating perspectives from additional stakeholder groups, including policymakers, HTA committee members, clinicians, and patient representatives, could further enrich understanding of how HTA processes operate across the full decision-making landscape.

## Data Availability

The raw data supporting the conclusions of this article will be made available by the authors, without undue reservation.

## Footnotes

1
https://www.sfee.gr

2
https://www.sfee.gr/o-sfee/meli

## References

[1] Morgan D Astolfi R. Health Spending Growth at Zero: Which Countries, Which Sectors are Most Affected? OECD Health Work Papers, No. 60. Paris: OECD Publishing (2013).

[2] Parmar D Stavropoulou C Ioannidis JPA. Health outcomes during the 2008 financial crisis in Europe: systematic literature review. BMJ. (2016) 354:i4588. doi: 10.1136/bmj.i4588, 27601477 PMC5013230

[3] Szczepański M. A Decade on from the Crisis: Main Responses and Remaining Challenges. European Parliamentary Research Service (EPRS) Briefing, PE 642.253. Luxembourg: Publications Office of the European Union (2019).

[4] OECD/European Observatory on Health Systems and Policies. Greece: Country Health Profile 2019 (State of Health in the EU). Paris: OECD Publishing (2019).

[5] Banta D. The development of health technology assessment. Health Policy. (2003) 63:121–32. doi: 10.1016/S0168-8510(02)00059-3, 12543525

[6] Drummond MF Sculpher MJ Claxton K Stoddart GL Torrance GW. Methods for the Economic Evaluation of Health Care Programmes. 4th ed. Oxford: Oxford University Press (2015).

[7] World Health Organization. Global Survey on Health Technology Assessment by National Authorities. Geneva: World Health Organization (2015).

[8] Hellenic Republic. (2018a). Law 4512/2018: Regulations for the implementation of the structural reforms of the economy program and other provisions [Rithmiseis gia tin efarmogi ton diarthotikon metarrithmisewn tou programmatos oikonomikis kai alles diatakseis]. Official Government Gazette (FEK) A’ 5.

[9] Hellenic Republic. (2018b). Approval of the internal regulation of the Evaluation and Reimbursement Committee for medicines for human use – Law 4512/2018 [Egkrisi tou esoterikou kanonismou leitourgias tis epitropis aksiologisis kai apozimiwsis farmakon anthropinis xrisis tou nomou 4512/2018]. Official Government Gazette (FEK) B’ 2768.

[10] Economou C. Kaitelidou D. Kentikelenis A. Maresso A. Sissouras A. (2015). The impact of the crisis on the health system and health in Greece. In: Maresso A. Mladovsky P. Thomson S., (eds.) Economic Crisis, Health Systems and Health in Europe: Country Experience (Observatory Studies Series, No. 41). Copenhagen: European Observatory on Health Systems and Policies. Available online at: https://www.ncbi.nlm.nih.gov/books/NBK447857/28837306

[11] Thomson S Figueras J Evetovits T Jowett M Mladovsky P Maresso A . Economic Crisis, Health Systems and Health in Europe: Impact and Implications for Policy (policy summary 12). Copenhagen: WHO Regional Office for Europe / European Observatory on Health Systems and Policies (2014). 28837306

[12] Mylonas C Dalakaki E Skroumpelos A Karokis A. Implementing the recovery and resilience fund initiative to reduce pharmaceutical expenditure clawback in Greece: the impact on pharmaceutical spending and on national economy. Value Health. (2022) 25:S246. doi: 10.1016/j.jval.2022.09.1207

[13] Athanasakis K Thireos E Geitona M Yfantopoulos J Kyriopoulos J. A proposal for the procedures and organization of health technology assessment in Greece. Arch Hellen Med. (2020) 37:439–44.

[14] Beletsi A Stefanou G Kourlaba G. Time from marketing authorization to reimbursement of medicines in Greece after the introduction of the health technology assessment process from July 2018 to April 2022. Value Health Reg Issues. (2023) 36:58–65. doi: 10.1016/j.vhri.2023.03.001, 37030032

[15] Chantzaras A Margetis A Kani C Koutsiouris V Bacopoulou F. Health technology assessment of medicinal products in Greece: a 5-year (2018–2023) review of timelines and productivity. Int J Technol Assess Health Care. (2024) 40:e40. doi: 10.1017/S0266462324000485, 39494839 PMC11563181

[16] DeJonckheere M Vaughn LM. Semistructured interviewing in primary care research: a balance of relationship and rigour. Family Med Commun Health. (2019) 7:e000057. doi: 10.1136/fmch-2018-000057, 32148704 PMC6910737

[17] Palinkas LA Horwitz SM Green CA Wisdom JP Duan N Hoagwood K. Purposeful sampling for qualitative data collection and analysis in mixed method implementation research. Admin Pol Ment Health. (2015) 42:533–44. doi: 10.1007/s10488-013-0528-y PMC4012002 24193818

[18] Gill P Stewart K Treasure E Chadwick B. Methods of data collection in qualitative research: interviews and focus groups. Br Dent J. (2008) 204:291–5. doi: 10.1038/bdj.2008.192, 18356873

[19] Kallio H Pietilä A-M Johnson M Kangasniemi M. Systematic methodological review: developing a framework for a qualitative semi-structured interview guide. J Adv Nurs. (2016) 72:2954–65. doi: 10.1111/jan.13031, 27221824

[20] Braun V Clarke V. Using thematic analysis in psychology. Qual Res Psychol. (2006) 3:77–101. doi: 10.1191/1478088706qp063oa

[21] Nowell LS Norris JM White DE Moules NJ. Thematic analysis: striving to meet the trustworthiness criteria. Int J Qual Methods. (2017) 16:1–13. doi: 10.1177/1609406917733847

[22] Elo S Kyngäs H. The qualitative content analysis process. J Adv Nurs. (2008) 62:107–15. doi: 10.1111/j.1365-2648.2007.04569.x, 18352969

[23] Byrne M. The concept of informed consent in qualitative research. AORN J. (2001) 74:401–3. doi: 10.1016/S0001-2092(06)61798-5, 11565159

[24] Kaiser K. Protecting respondent confidentiality in qualitative research. Qual Health Res. (2009) 19:1632–41. doi: 10.1177/1049732309350879, 19843971 PMC2805454

[25] O’Brien BC Harris IB Beckman TJ Reed DA Cook DA. Standards for reporting qualitative research: a synthesis of recommendations. Acad Med. (2014) 89:1245–51. doi: 10.1097/ACM.0000000000000388, 24979285

[26] Francis JJ Johnston M Robertson C Glidewell L Entwistle V Eccles MP . What is an adequate sample size? Operationalising data saturation for theory-based interview studies. Psychol Health. (2010) 25:1229–45. doi: 10.1080/08870440903194015, 20204937

[27] Guest G Namey EE Mitchell ML. Collecting Qualitative Data: A Field Manual for Applied Research. Thousand Oaks, CA: SAGE Publications (2013).

[28] Hennink M Kaiser BN. Sample sizes for saturation in qualitative research: a systematic review of empirical tests. Soc Sci Med. (2022) 292:114523. doi: 10.1016/j.socscimed.2021.114523, 34785096

[29] Tong A Sainsbury P Craig J. Consolidated criteria for reporting qualitative research (COREQ): a 32-item checklist for interviews and focus groups. Int J Qual Health Care. (2007) 19:349–57. doi: 10.1093/intqhc/mzm042, 17872937

[30] Hellenic Republic. (2019). Law 4633/2019, Article 22: Evaluation criteria and methodology.

[31] Chootipongchaivat S. Tritasavit N. Luz A. Teerawattananon Y. Tantivess S. (2015) Factors conducive to the development of health technology assessment in Asia: Impacts and policy options (Policy Brief, Vol. 4, No. 2) Manila WHO Regional Office for the Western Pacific/Asia Pacific Observatory on Health Systems and Policies / Prince Mahidol Award Foundation

[32] Mbau R Vassall A Gilson L Barasa E. Factors influencing institutionalization of health technology assessment in Kenya. BMC Health Serv Res. (2023) 23:681. doi: 10.1186/s12913-023-09673-4, 37349812 PMC10288787

[33] Suharlim C Kumar R Salim J Mehra M Gilmartin C Amaris Caruso A . Exploring facilitators and barriers to introducing health technology assessment: a systematic review. Int J Technol Assess Health Care. (2022) 38:e5. doi: 10.1017/S0266462321000623 36317684

[34] Wang Y Qiu T Zhou J Francois C Toumi M. Which criteria are considered and how are they evaluated in health technology assessments? A review of methodological guidelines used in Western and Asian countries. Appl Health Econ Health Policy. (2021) 19:281–304. doi: 10.1007/s40258-020-00634-0, 33426626

[35] Vogler S Haasis MA Dedet G Lam J Bak Pedersen H. Medicines Reimbursement Policies in Europe. Copenhagen: WHO Regional Office for Europe (2018).

[36] Oortwijn W Husereau D Abelson J Barasa E Bayani DD Santos VC . Designing and implementing deliberative processes for health technology assessment: a good practices report of a joint HTAi/ISPOR task force. Int J Technol Assess Health Care. (2022) 38:e37. doi: 10.1017/S0266462322000198 35656641 PMC7613549

[37] Hailey D. Toward transparency in health technology assessment: a checklist for HTA reports. Int J Technol Assess Health Care. (2003) 19:1–7. doi: 10.1017/S0266462303000011, 12701934

[38] National Institute for Health and Care Excellence. Policy on Conflicts of Interest (Version 2.5). London: NICE (2017).

[39] Wale JL Chandler D Collyar D Hamerlijnck D Saldana R Pemberton-Whitley Z. Can we afford to exclude patients throughout health technology assessment? Front Med Technol. (2022) 3:796344. doi: 10.3389/fmedt.2021.796344, 35146487 PMC8821945

[40] Chamova J. Mapping of HTA National Organisations, Programmes and Processes in EU and Norway. Luxembourg: Publications Office of the European Union (2017).

[41] Bertram MY Lauer JA Stenberg K Edejer TTT. Methods for the Economic Evaluation of Health Care Interventions for Priority Setting in the Health System: An Update From WHO CHOICE. Int J Health Policy Manag. (2021) 10:673–677. doi: 10.34172/ijhpm.2020.244, 33619929 PMC9278384

