# Supplementary material for: Strengthening health technology assessment in Greece: industry-identified barriers and recommendations
Source: Front Public Health. 2026 Mar 10;14:1790128. doi: 10.3389/fpubh.2026.1790128 (PMC13010272; doi:10.3389/fpubh.2026.1790128)
Supplement: Supplementary file 1 [file Supplementary_file_1.docx]

Supplementary Material

Strengthening Health Technology Assessment in Greece:
*Industry-identified barriers and recommendations*

Papageorgiou George^1^, Karampli Eleftheria^2^, Athanasakis Kostas^3^

# Introduction

Health Technology Assessment (HTA) has been increasingly adopted internationally over the past decades as a policy tool to support evidence-informed decision-making. In Greece, the Committee for the Evaluation and Reimbursement of Medicines for Human Use (hereafter, HTA Committee) was established in 2018 alongside the Price Negotiation Committee (hereafter, Negotiation Committee) through Law 4512/2018 (Banta D. et al., 2019; Hellenic Republic, 2018a).

This supplementary material presents additional findings derived from the perspectives of pharmaceutical industry stakeholders participating in the study. Executives were invited to share views based on their professional experience with the Greek HTA and negotiation pathway, as well as comparative insights drawn from other healthcare systems. The material focuses on industry-identified challenges and recommendations, complementing the main manuscript by providing further thematic detail.

# Highlights of the study

1. Seventeen Market Access Heads/Directors identified multiple barriers and proposed recommendations to strengthen Health Technology Assessment (HTA) in Greece.
2. Transparency, procedural guidance, and digitalization were perceived as key enablers for improving HTA processes.
3. Participants emphasized the importance of adequate staffing, expertise, and continuous training for effective HTA implementation.
4. Participants highlighted limited stakeholder involvement as a challenge to the evolution of the HTA system.
5. Several participants expressed the view that linking reimbursement outcomes more closely to clinical value could improve HTA decision-making.

# HTA process in Greece

According to its internal regulations, HTA Committee is responsible for evaluating the drugs with clear criteria and methodological rigor, giving an appraisal based on 4 criteria:

1. *clinical benefit,*
2. *the comparison with already available reimbursed treatments,*
3. *the degree of reliability of clinical study data and*
4. *the cost-effectiveness ratio.*

Then, it refers them to the Negotiation Committee to be evaluated for their impact on the budget, if any. Although budget impact assessment is described in the legislation as part of the HTA Committee’s remit, in practice this activity is primarily undertaken by the Negotiation Committee. After the end of the negotiation with the Marketing Authorization Holder (MAH), the Negotiation Committee informs the HTA about its own recommendation, so that HTA Committee, in turn, communicates the final appraisal to the Minister of Health (Hellenic Republic, 2018b).

# MAH’s application and supporting documents

The evaluation process begins with MAH’s application, and HTA Committee’s secretariat checks the supporting documents described in supplementary material table 1.

The HTA Committee consists of eleven (11) members including the President and the Vice President. According to the legislation, its members should have expertise in at least one of the following specialties and there should be diversity among them: (clinical) pharmacology, clinical studies, economic evaluation (cost-effectiveness analysis), (bio)statistics, pharmaco-epidemiology, pharmaco-economics, preparation of treatment protocols (Hellenic Republic, 2018b).

For the participation in HTA Committee, either as a regular member or external expert, they all must have declared about themselves and their close relatives (second degree) no conflict of interests with the pharmaceutical industry (Hellenic Republic, 2018b).

Finally, according to the legislation, HTA Committee may invite representatives from patient associations or medical societies, to include their views in its final decision (Hellenic Republic, 2018a).

# Thematic Analysis results

## Theme 8: The role of Associations of pharmaceutical companies in Health Technology Assessment

*This theme reflects participants’ perceptions of the role of pharmaceutical industry associations in the HTA process and their views on how these organizations could contribute to improving evaluation procedures in Greece.*

The great majority declared dissatisfaction with the actions of the Associations with only a very small number to have expressed satisfaction. As associations they refer mainly to SfEE (Hellenic Association of Pharmaceutical Companies) and Pharma Innovation Forum (PIF), however there is also a report on the Panhellenic Union of Pharmaceutical Industry (PEF). The topics touched on are the conflict of interests between multinational and Greek companies, the scope for strengthening the role and influence of the associations, the way of submitting and monitoring proposals, the industry's own knowledge of technology assessment issues, the orientation of strategies in discount issues, etc.

Participant 13 says: *“There are proposals, there were some with good points, but I feel that S.f.E.E., PIF etc. they don't have the ability to influence."*

However, a point of view, which is common to several participants, is interesting, with strong self-criticism about the issue of adequacy (knowledge and experience) of the industry itself regarding HTA issues.

Participant 3 states: *“In the industry we have very few health economists, few in market access and very few experienced. It's not that they don't know but we do. Neither of us really knows.”*

## Theme 9: The role of pharmaceutical associations in improving evaluation processes

### *The adoption of a common European framework for clinical evaluations*

*This theme reflects participants’ perceptions and expectations regarding the adoption of a common European framework for clinical evaluations.*

Most of the participants, although generally positive with the common clinical assessments, listed a few concerns such as: the next day of local assessments, deviations, and application of the framework for all markets, local data (epidemiological data etc.), additional delays, overlapping of responsibilities with the EMA, savings, reforms required in all markets to adopt, etc.

Participant 7 states: *" I think that the European Medicines Agency (EMA) has done most of the work. So, what more to analyze? We know that all of this will become time-consuming, I don't think that the local HTAs will stop, it will just add another layer of assessment that will delay the innovation to re-examine the same things."*

There were also some views raised the issue that countries with well-structured HTA organizations will be reluctant to contribute to this initiative because of their possible weakening and the quality and speed of the results that will be issued by the joint clinical evaluations.

Finally, a small percentage declared positive towards this transition as it will lead to a series of improvements that will provide a significant benefit to the health system, such as reducing delays, saving resources, and simplifying procedures.

### *Necessity of a national assessment framework*

Many participants mentioned the importance of supporting the assessment with local data, which differ from market to market, as do patient and system needs.

Participant 12 states: *"I think that at the clinical level in general in Europe things are converging. However, this can never replace the local factor, in terms of market conditions, because its parts are related to parameters not clinical, social, fiscal, etc.*

# Interview guide

1. HTA implementation happened in 2018 in Greece. What were your expectations before the establishment of it?
2. To what extent were they fulfilled and to what extent were they not?
3. In which areas were these fulfilled, in which not (degree of satisfaction)
4. According to the legislation there are basic evaluation criteria for a new health technology. Do you think that the evaluation criteria during the HTA procedure are met?
5. How would you evaluate the existing organizational structure of HTA in Greece?
6. Do you think that the implementation of the Government's commitment to the establishment of an HTA Organization would improve the relevant procedures?
7. How do you judge the prospect of a common European HTA regulation for the purpose of joint clinical assessments?
8. What is your opinion on the role of pharmaceutical industry associations in improving HTA procedures in Greece?
9. What is the role of patients in HTA Committee?
10. If you could share one or two recommendations to improve the HTA procedures in Greece, what would they be?

*This supplementary material complements the main manuscript by providing additional thematic detail and illustrative quotations that could not be included in the main text due to space constraints.*

# Supplementary Material Tables

**Supplementary Material Table 1:** *Prerequisite supporting documents for submitting an evaluation file ((Hellenic Republic, 2019; Hellenic Republic, 2021).*

| **Submission Requirements** | **Details** | **Exclusions** |
| --- | --- | --- |
| **Marketing Authorization** |  |  |
| **Treatment limitations** |  |  |
| **Submission forms** |  |  |
| **Evaluation file** | *It includes all the necessary elements and data that fall into the process (e.g., budget impact model, cost effectiveness model, etc.) as well as the evaluation criteria.* |  |
| **The opinion and the reasoning behind it, from the organizations European Medicines Agency and Committee for Medicinal Products for Human Use (CHMP).** |  |  |
| **Epidemiological studies etc.** | *They relate to the technology and/or the target population of the intervention.* |  |
| **Evaluation that has resulted from five other Health Technology Assessment Organizations/Committees of specific European countries.** | - *External Criteria: five out of 11 or just hereinafter 5/11.* - *Five countries out of a predetermined eleven countries which are: France, Italy, Germany, Spain, Denmark, Austria, Belgium, Portugal, Netherlands and from the Nordic countries Finland and Sweden.* | *Orphan drugs, clones, biosimilars, well-established drugs and vaccines.* |
| **Completion of the evaluation criteria** |  |  |
| **Summary of all the above in the form of a report** |  |  |
| **Assessment fee deposit** | - *€5.000 for all drugs* - *€3,000 for generics and fixed combination* |  |

**Supplementary Material Table 2:** *Consolidated Criteria for Reporting Qualitative Research (COREQ) checklist*

| **Item** | **COREQ item** | **Where addressed/ Notes** |
| --- | --- | --- |
| **Research team and reflexivity** | | |
| 1 | Interviewer / facilitator | All interviews were conducted by a single researcher.  *(Methods – Conducting the interviews)* |
| 2 | Credentials | The interviewer had professional experience in health policy, pharmaceutical policy and market access.  *(Methods – Data analysis)* |
| 3 | Occupation | Professional familiarity with medicines reimbursement and market access processes in Greece.  *(Methods – Data analysis)* |
| 4 | Gender | Not reported. |
| 5 | Experience and training | Professional experience in pharmaceutical policy and market access research.  *(Methods – Data analysis)* |
| 6 | Relationship established | Some participants were approached through professional networks.  *(Methods – Description of participants)* |
| 7 | Participant knowledge of the interviewer | Participants were informed about the study purpose and researcher role through the consent process.  *(Methods – Ethics and consent)* |
| 8 | Interviewer characteristics | Reflexive notes were maintained throughout data collection and analysis to minimize the influence of prior assumptions.  *(Methods – Data analysis)* |
| Domain 2: Study Design | | |
| 9 | Methodological orientation and theory | Exploratory qualitative design using thematic analysis.  *(Methods – Data analysis)* |
| 10 | Sampling | Purposive and snowball sampling.  *(Methods – Description of participants)* |
| 11 | Method of approach | Participants were approached through professional networks and direct outreach. *(Methods – Description of participants)* |
| 12 | Sample size | Seventeen participants were interviewed. *(Methods – Conducting the interviews)* |
| 13 | Non-participation | Information on refusals or non-participation was not recorded. |
| 14 | Setting of data collection | Online interviews conducted via Teams and Zoom.  *(Methods – Conducting the interviews)* |
| 15 | Presence of non-participants | No non-participants were present during the interviews. |
| 16 | Description of sample | Senior Market Access executives from pharmaceutical companies operating in Greece. *(Methods – Description of participants)* |
| **Data collection** | | |
| 17 | Interview guide | Semi-structured interview guide used and pilot tested.  *(Methods – Data collection; Supplementary Material – Interview Guide)* |
| 18 | Repeat interviews | Repeat interviews were not conducted. |
| 19 | Audio/visual recording | Interviews were audio-recorded with participant consent.  *(Methods – Conducting the interviews)* |
| 20 | Field notes | Reflexive notes were maintained throughout data collection and analysis.  *(Methods – Data analysis)* |
| 21 | Duration | Interviews lasted approximately one hour. *(Methods – Conducting the interviews)* |
| 22 | Data saturation | Data saturation was reached at the 17th interview.  *(Methods – Conducting the interviews)* |
| 23 | Transcripts returned | Transcripts were not returned to participants for comment or correction. |
| Domain 3: Analysis and findings | | |
| 24 | Number of data coders | Two researchers independently coded the first five transcripts; subsequent coding was conducted by the main researcher.  *(Methods – Data analysis)* |
| 25 | Description of the coding tree | Codes were iteratively grouped into higher-order themes through an inductive process, with themes reviewed for internal coherence and conceptual distinction.  *(Methods – Data analysis)* |
| 26 | Derivation of themes | Themes were derived using a combination of inductive and deductive approaches.  *(Methods – Data analysis)* |
| 27 | Software | No qualitative data analysis software was used. |
| 28 | Participant checking | Participants did not provide feedback on the findings. |
| 29 | Quotations presented | Participant quotations are presented to illustrate themes.  *(Results section)* |
| 30 | Data and findings consistent | Clear linkage between data excerpts and identified themes.  *(Results section)* |
| 31 | Clarity of major themes | Major themes are clearly presented and structured.  *(Results section)* |
| 32 | Clarity of minor themes | Minor and divergent perspectives are reported through sub-themes and quotations.  *(Results section)* |

# Supplementary Material References

Banta, D., Kristensen, F.B. and Jonsson, E. (2009) ‘A history of health technology assessment at the European level’, *International Journal of Technology Assessment in Health Care*, 25(S1), pp. 68–73.

Tong, A., Sainsbury, P. and Craig, J. (2007) ‘Consolidated criteria for reporting qualitative research (COREQ): a 32-item checklist for interviews and focus groups’, International Journal for Quality in Health Care, 19(6), pp. 349–357. doi:10.1093/intqhc/mzm042.

Hellenic Republic (2018a) *Law 4512/2018: Arrangements for the implementation of the Structural Reforms of the Economic Adjustment Program and other provisions*, Government Gazette (FEK) A’.

Hellenic Republic (2018b) *Approval of the internal regulation of the Committee for the Evaluation and Reimbursement of Medicines for Human Use (Law 4512/2018)*, Government Gazette (FEK) B’, No. 2768.

Hellenic Republic (2019) *Law 4633/2019, Article 22: Evaluation criteria and methodology*.

Hellenic Republic (2021) *Determination of the one-time fee for the evaluation of medicines by the Committee for the Evaluation and Reimbursement of Medicines for Human Use*, Government Gazette (FEK) B’, No. 1490.

# Abbreviations

*In the order in which they appear in the supplementary material.*

1. HTA: *Health Technology Assessment*
2. HTA Committee: *Committee for the Evaluation and Reimbursement of Medicines for Human Use (in Greece)*
3. Negotiation Committee: *Price Negotiation Committee (in Greece)*
4. MAH(s): *Marketing Authorization Holder(s)*
5. SfEE: *Hellenic Association of Pharmaceutical Companies*
6. PIF: *Pharma Innovation Forum*
7. PEF*: Panhellenic Union of Pharmaceutical Industry*
8. EMA: *European Medicines Agency*
9. CHMP: *Committee for Medicinal Products for Human Use*

*All findings presented in this supplementary material reflect participants’ views and experiences and should be interpreted as stakeholder perceptions rather than objective evaluations of system performance.*
